# Nurse Practitioner’s Clinical Triggers for Referral to Other 3D Team Members

**DOI:** 10.1093/geroni/igaa057.2680

**Published:** 2020-12-16

**Authors:** Shawn Ladda

**Affiliations:** UConn Health, Farmington, Connecticut, United States

## Abstract

This presentation features how 3D Team nurse practitioners (NP) use results of clinical assessments to determine whether older adults and caregivers enrolled in the study are referred to other Team members; these assessment results are called “clinical triggers”. Other team members who receive referrals based on NP-generated clinical triggers include: Licensed Clinical Social Workers, who deliver Problem Solving Therapy to older adults with significant depressive symptoms; Occupational Therapists, who deliver an evidence-based dementia care intervention; Physical Therapists, who deliver an adapted Otago exercise program; Registered Dietician, who provides nutrition and dietary instruction; and Community Health Educator, who provides community resource information to address social determinants of health. All clinical triggers will be detailed in this presentation, along with a description of each intervention delivered by other team members except the Community Health Educator. Case studies will be presented to illustrate how study participants receive multiple interventions from the 3D Team.

